# Severe anaemia is associated with a higher risk for preeclampsia and poor perinatal outcomes in Kassala hospital, eastern Sudan

**DOI:** 10.1186/1756-0500-4-311

**Published:** 2011-08-26

**Authors:** AbdelAziem A Ali, Duria A Rayis, Tajeldin M Abdallah, Mustafa I Elbashir, Ishag Adam

**Affiliations:** 1 Faculty of Medicine, Kassala University, Kassala, Sudan; 2 Faculty of Medicine, University of Khartoum, Khartoum, Sudan

## Abstract

**Background:**

Anaemia during pregnancy is major health problem. There is conflicting literature regarding the association between anaemia and its severity and maternal and perinatal outcomes.

**Methods:**

This is a retrospective case-control study conducted at Kassala hospital, eastern Sudan. Medical files of pregnant women with severe anaemia (haemoglobin (Hb) < 7 g/dl, n = 303) who delivered from January 2008 to December 2010 were reviewed. Socio-demographic and obstetric data were analysed and compared with a similar number of women with mild/moderate anaemia (Hb = 7-10.9 g/dl, n = 303) and with no anaemia (Hb > 11 g/dl, n = 303). Logistic regression analysis was performed separately for each of the outcome measures: preeclampsia, eclampsia, preterm birth, low birth weight (LBW) and stillbirth.

**Results:**

There were 9578 deliveries at Kassala hospital, 4012 (41.8%) women had anaemia and 303 (3.2%) had severe anaemia. The corrected risk for preeclampsia increased only in severe anaemia (OR = 3.6, 95% CI: 1.4-9.1, *P *= 0.007). Compared with women with no anaemia, the risk of LBW was 2.5 times higher in women with mild/moderate anaemia (95% CI: 1.1-5.7), and 8.0 times higher in women with severe anaemia (95% CI: 3.8-16.0). The risk of preterm delivery increased significantly with the severity of anaemia (OR = 3.2 for women with mild/moderate anaemia and OR = 6.6 for women with severe anaemia, compared with women with no anaemia). The corrected risk for stillbirth increased only in severe anaemia (OR = 4.3, 95% CI: 1.9-9.1, *P *< 0.001).

**Conclusions:**

The greater the severity of the anaemia during pregnancy, the greater the risk of preeclampsia, preterm delivery, LBW and stillbirth. Preventive measures should be undertaken to decrease the prevalence of anaemia in pregnancy.

## Background

Anaemia during pregnancy is a major public health problem, especially in developing countries [1]. It affects 41.8% of pregnant women globally, with the highest prevalence in Africa [2]. There is however significant variation in the prevalence of anaemia both within and between countries, necessitating a need for local data to help to improve preventive programmes. Anaemia during pregnancy, especially severe anaemia, is associated with increased maternal morbidity and mortality and contributes to 20% of the maternal mortality in Africa [1,3,4]. Anaemia during pregnancy is associated with a negative impact on both the woman and neonate. Fetal anaemia, low birth weight (LBW), preterm birth and stillbirth have been associated with anaemia [4-9].

There is conflicting literature regarding the association between anaemia and perinatal outcomes. Some recent studies [8,10] have demonstrated a strong association between anaemia and adverse perinatal outcomes such as preterm delivery and LBW, while other previous studies found no association [11,12]. A meta-analysis showed that anaemia during early pregnancy, but not during late pregnancy, is associated with slightly increased risk of preterm delivery and LBW [13]. Many studies have used different definitions and were undertaken in areas with a low prevalence of anaemia [13]. There is therefore insufficient information to conclusively assess the effect of maternal anaemia on maternal and perinatal outcomes. Furthermore, most studies were not able to study anaemia according to its severity.

In Kassala hospital, eastern Sudan we have recently reported a high prevalence of obstetric complications [14], high maternal mortality rate and high prevalence of anaemia including severe anaemia among pregnant women [15,16]. The current study was therefore conducted in Kassala hospital to investigate if severe anaemia is a risk factor for preeclampsia, eclampsia, preterm birth, LBW and stillbirth.

## Methods

Kassala, eastern Sudan, is an area of 42282 km^2 ^located nearly 600 km from the capital city Khartoum with a population of 1.8 million including 440491 women of reproductive age. Kassala hospital provides tertiary care for women who receive antenatal care at the hospital, as well as for referrals from other clinics and hospitals and for women who live close to the hospital facility. All women with risk factors or obstetric complications are referred to this hospital. However, the referral criteria are not strictly adhered to and many patients with no significant complications also deliver at the hospital.

The medical files of women with severe anaemia who delivered at Kassala maternity hospital during the 3 year period from January 2008 to December 2010 were retrospectively retrieved. The data of severely anaemic cases were compared with similar numbers of cases with mild/moderate anaemia with no anaemia. The control group (no anaemia) were women with file numbers following the cases of severe anaemia.

Data retrieved included socio-demographic characteristics (age, parity, residence, education and antenatal care), maternal outcomes (preterm birth, preeclampsia, eclampsia and heart failure) and neonatal outcomes (preterm birth, LBW and stillbirth). Only women with singleton pregnancies were included.

Maternal anaemia was defined when haemoglobin (Hb) was < 11 g/dl and was classified as mild/moderate (7-10.9 g/dl) or severe (< 7 g/dl) [1]. Preeclampsia was defined as diastolic blood pressure ≥ 90 mmHg and/or systolic blood pressure ≥ 140 mmHg recorded on 2 occasions 4 hours apart, plus dipstick proteinuria ≥ 2+, after the 20th week of gestation in a previously normotensive woman. Eclampsia, a severe complication of preeclampsia, was defined as the new onset of seizures in a woman with preeclampsia. Gestational age in weeks was calculated from the first day of the last menstrual period. Preterm delivery was defined as delivery before 37 completed weeks of gestation. LBW was defined as < 2.5 kg. Stillbirth was defined as delivery of a dead infant after 24 weeks of gestation.

### Statistics

Data were entered into a computer database using SPSS software (SPSS Inc., Chicago, IL, USA, version 16.0). Comparisons between categorical variables were made using the χ^2 ^test, and between quantitative variables using the independent *t*-test or one-way ANOVA. A *P *value of less than 0.05 was considered statistically significant. Logistic regression analysis was performed separately for preeclampsia, eclampsia, preterm birth, LBW and stillbirth to evaluate the effects of both mild/moderate and severe maternal anaemia. Odds ratios (ORs) and their corresponding 95% confidence intervals (CIs) were calculated, using women with no anaemia as the reference category. The corrected ORs were calculated after removing the confounders in each model (age, parity, education, residence and antenatal care). Preeclampsia and eclampsia were regarded as confounders when we investigated the risk for perinatal outcomes.

### Ethics

The study received ethical approval from the Health Research Board at the Ministry of Health, Kassala, Sudan.

## Results

### Patient characteristics

There were 9578 deliveries at Kassala hospital during the study period. Of these, 4012 (41.8%) women had anaemia and 303 (3.2%) had severe anaemia at the time of delivery. Age and parity were not significantly different between women with no anaemia, women with mild/moderate anaemia and women with severe anaemia. A significantly higher number of women with severe anaemia had lower antenatal care attendance, lower level of education and rural residence, table 1.

**Table 1:** Socio-demographic characteristics of the investigated women

Variable	Normal *(N *= 303)	Mild/moderate anaemia (*N *= 303)	Severe anaemia (*N *= 303)	*P*
Age, years	32.2(6.1)	33.2 (5.0)	33 (6.0)	0.1
Parity	3.2(2)	3.2(2.4)	3.4(2.1)	0.3
Rural residence	113 (37.3%)	231(76.2%)	246 (81.2%)	0.001
lack of antenatal care	98 (32.3%)	163 (53.7%)	229 (75.5%)	< 0.001
Education < secondary level	73 (24%)	173 (57%)	176 (58%)	< 0.001

### The association between anaemia and preeclampsia and eclampsia

The prevalence of preeclampsia and eclampsia was significantly higher in women with severe anaemia (8.2% and 3.3%, respectively), table 2. The corrected risk for preeclampsia (OR = 3.6, 95% CI: 1.4-9.1, *P *= 0.007) increased only in severe anaemia, table 3. Logistic regression analysis showed that maternal age was also a risk factor for preeclampsia, with a higher risk in women aged < 20 years (OR = 7.6, 95% CI: 2.9-19.9) and in women aged > 35 years (OR = 10.2, 95% CI: 3.2-32.2). The risk for eclampsia was not increased in women with anaemia, table 3. There were three maternal deaths due to heart failure in the group with severe anaemia.

**Table 2:** Maternal and perinatal outcomes in the investigated women*

Variable	non-anaemic (N = 303)	mild/moderate anaemia (N = 303)	severe anaemia (N = 303)	P
Preeclampsia	7(2.3%)	14(4.6%)	25(8.2)	0.004
Eclampsia	2(0.7%)	4(1.3%)	10(3.3%)	0.03
Preterm birth	7(2.3)	11(3.6)	35(11.5)	< 0.001
low birth weight	10(3.3%)	23(7.5)	63(20.7)	< 0.001
Stillbirth	9(2.9)	21(6.9)	42(13.8)	< 0.001

*Data were shown as n (%) and the *P *value is for X^2 ^test comparing the proportions in the subgroups.

**Table 3:** Corrected odds ratio and 95% confidence interval for anaemia and pregnancy outcome in Kassala Hospital, Eastern Sudan

Variables	Mild/moderate anaemia	Severe anaemia
	**OR (95% CI)*, P***	**OR (95% CI), *P***
	
Preeclampsia	1.6 (0.8-3.4), 0.1	3.6(1.4-9.1), 0.007
Eclampsia	2.3(0.7-7.8), 0.1	4.0(0.7-20.9), 0.09
Preterm delivery	3.2(1.5-6.6),0.001	6.6(2.7-16.3),< 0.001
Low birth weight	2.5 (1.1-5.7),0.02	8.0(3.8-16.0),< 0.001
Stillbirth	1.8 (0.7-4.4),0.1	4.3(1.9-9.1), < 0.001

### The association between anaemia and perinatal outcomes

Women with severe anaemia delivered infants with a significantly lower birth weight than women with mild/moderate anaemia and women with no anaemia (mean [standard deviation] = 2.9 [0.7] vs. 3.1 [0.6] vs. 3.3 [0.5] kg, *P *< 0.001) and with a lower gestational age (37.3 [8.4] vs. 38.2 [7.1] vs. 39.5 [6.3], *P *= 0.001). The prevalence of preterm delivery and LBW was significantly higher in women with anaemia, table 2 and increased with the severity of anaemia. Compared with women with no anaemia, the risk of LBW was 2.5 times higher (95% CI: 1.1-5.7) in women with mild/moderate anaemia and 8.0 times higher (95% CI: 3.8-16.0) in women with severe anaemia. Compared with women with no anaemia, the risk of preterm delivery increased significantly with the severity of anaemia (mild/moderate anaemia OR = 3.2, 95% CI: 1.5-6.6; severe anaemia OR = 6.6, 95% CI: 2.7-16.3), table 3. The corrected risk for stillbirth increased only in severe anaemia (OR = 4.3, 95% CI: 1.9-9.1, *P *< 0.001), table 3.

## Discussion

This study revealed that maternal anaemia is severe problem in Kassala. According to the World Health Organization, a severe public health problem exists if the prevalence of anaemia is ≥ 40% in any group [1]. Our data demonstrate a need to re-evaluate or strengthen the current strategies to decrease the prevalence of anaemia among women of reproductive age in this setting. Anaemia has previously been found to affect pregnant women in eastern Sudan regardless of their age and parity [17], which is confirmed in this study. Our data showed that women with severe anaemia were less educated, and had a lower rate of antenatal attendance and a higher rate of rural residency. We also recently observed an association between education level and antenatal care, and an influence of both education and antenatal care on maternal mortality [15].

In the current study, women with severe anaemia had a 3.6 times higher risk of preeclampsia than women with no anaemia. It was recently observed that 17 (17.7%) of 97 women with severe anaemia had gestational hypertension or preeclampsia and 2 (2.1%) had eclampsia [10]. However, it might be difficult to reach a firm conclusion from this report since the authors did not mention the incidence of these events in women with mild/moderate anaemia or with no anaemia. The susceptibility of women with severe anaemia to preeclampsia could be explained by a deficiency of micronutrients and antioxidants. Recent results indicate that reduction in serum levels of calcium, magnesium and zinc during pregnancy might be possible contributors to the development of preeclampsia [18]. We have recently observed a high prevalence of both anaemia (including severe anaemia) and micronutrient deficiency in the same hospital [16]. It might be difficult to determine if severe anaemia was the cause or effect of preeclampsia/eclampsia in this study as the anaemia was diagnosed at admission and may have been a consequence of the disease process (haemolysis in HELLP syndrome). A longitudinal study with a large sample size is needed to explore whether severe anaemia is a cause or effect of preeclampsia. There may, however, be ethical issues with such a study as severe anaemia is a medical emergency which should be prevented and treated immediately. Poor maternal and perinatal outcomes in cases of anaemia associated with malaria have recently been documented in some regions of Sudan including Kassala [19]. It appears probable that the severe anaemia caused by malaria is a major factor in those outcomes. Previous studies have indicated that malaria increases the risk of hypertensive disorder during pregnancy [20]. Placental histology is the gold standard for the diagnosis of malaria during pregnancy, and is preferred over the peripheral blood film, which has many abnormalities in this setting [21].

Interestingly, earlier studies have reported a higher incidence of preeclampsia and an increased incidence of hypertensive disorders in women with high Hb levels (13.3 g/dl and 12.5 g/dl) than in those with normal levels [22,23]. Murphy et al. (1986) reported that, in primiparas, the frequency of hypertension ranged from 7% with Hb < 10.5 g/dl to 42% with Hb > 14.5 g/dl [24]. The increased incidence of preeclampsia in pregnant women with high Hb levels could be explained by the toxic effects of methaemoglobin-derived haeme deposition on the vascular endothelium and consequent atherosclerosis [24,25]. Atherosclerotic blood vessels were commonly seen in the placental beds of preeclamptic pregnancies [25,26].

The risk of preterm birth, LBW and stillbirth was higher in anaemic women, and increased with the severity of anaemia. This supports previous observations from Sudan as well as from other African countries [5,7,8]. LBW is one of the major causes of the 4 million neonatal deaths per year in developing countries. Neonatal deaths account for 38% of child deaths under the age of 5 years [27]. If the Millennium Development Goal to reduce the number of child deaths under the age of 5 years by two-thirds by 2015 is to be achieved, a substantial reduction in neonatal deaths is required. Reducing the incidence of LBW births is thus vital, and addressing maternal nutrition to prevent anaemia may a useful strategy in this setting [1,5].

One of the limitations of this study is the difficulty in dissecting preterm birth and LBW. It is difficult and inaccurate to draw conclusions regarding preterm birth and LBW when gestational age is based on the last menstrual period. LBW is highly correlated with gestational age at delivery and both are also correlated with other pregnancy complications such as preeclampsia. It is therefore difficult to draw conclusions regarding these complications when there are no data on gestational age at delivery and whether the preterm delivery was induced (as in preeclampsia/eclampsia) or spontaneous. The other limitation of this study is the weakness of retrospective methods of data collection with respect to the quality of records, the availability of comprehensive records and recollection bias.

## Conclusions

The greater the severity of the anaemia in pregnancy, the greater the risk of preeclampsia, preterm delivery, LBW and stillbirth. Preventive measures should be undertaken to decrease the prevalence of anaemia in pregnancy.

## Competing interests

The authors declare that they have no competing interests.

## Authors' contributions

AAA, DER and IA undertook the data collection and participated in the statistical analysis. TMA and MIE coordinated the study and participated in study design, statistical analysis and drafting of the manuscript. All authors read and approved the final version. AAA and IA are the guarantors for the paper.
